# Effective Chemoimmunotherapy with Anti-TGFβ Antibody and Cyclophosphamide in a Mouse Model of Breast Cancer

**DOI:** 10.1371/journal.pone.0085398

**Published:** 2014-01-09

**Authors:** Xin Chen, Yuan Yang, Qiong Zhou, Jonathan M. Weiss, OlaMae Zack Howard, John M. McPherson, Lalage M. Wakefield, Joost J. Oppenheim

**Affiliations:** 1 Basic Science Program, Leidos Biomedical Research, Inc., Frederick National Laboratory, Frederick, Maryland, United States of America; 2 Laboratory of Cancer Biology and Genetics, Center for Cancer Research, National Cancer Institute, Frederick, Maryland, United States of America; 3 Laboratory of Molecular Immunoregulation, Center for Cancer Research, National Cancer Institute, Frederick, Maryland, United States of America; 4 Laboratory of Experimental Immunology, Center for Cancer Research, National Cancer Institute, Frederick, Maryland, United States of America; 5 Genzyme Corporation, Framingham, Massachusetts, United States of America; Ohio State University, United States of America

## Abstract

TGFβ is reportedly responsible for accumulation of CD4^+^Foxp3^+^ regulatory T cells (Tregs) in tumor. Thus, we treated mouse 4T1 mammary carcinoma with 1D11, a neutralizing anti-TGFβ (1,2,3) antibody. The treatment delayed tumor growth, but unexpectedly increased the proportion of Tregs in tumor. In vitro, 1D11 enhanced while TGFβ potently inhibited the proliferation of Tregs. To enhance the anti-tumor effects, 1D11 was administered with cyclophosphamide which was reported to eliminate intratumoral Tregs. This combination resulted in long term tumor-free survival of up to 80% of mice, and the tumor-free mice were more resistant to re-challenge with tumor. To examine the phenotype of tumor infiltrating immune cells, 4T1-tumor bearing mice were treated with 1D11 and a lower dose of cyclophosphamide. This treatment markedly inhibited tumor growth, and was accompanied by massive infiltration of IFNγ-producing T cells. Furthermore, this combination markedly decreased the number of splenic CD11b^+^Gr1^+^ cells, and increased their expression levels of MHC II and CD80. In a spontaneous 4T1 lung metastasis model with resection of primary tumor, this combination therapy markedly increased the survival of mice, indicating it was effective in reducing lethal metastasis burden. Taken together, our data show that anti-TGFβ antibody and cyclophosphamide represents an effective chemoimmunotherapeutic combination.

## Introduction

It has been proposed that breast cancer is a naturally immunogenic tumor, since tumor antigen specific immunity can be detected in breast cancer patients, and tumor-reactive T cells are known to localize to the breast tumor microenvironment [1], [2]. How such tumor-reactive T cells can be sufficiently activated and expanded to eradicate cancer is a key issue in devising effective immunotherapy. One approach is to overcome the mechanisms of peripheral tolerance exploited by breast tumors for immune evasion [3]. CD4^+^FoxP3^+^ regulatory T cells (Tregs) and Gr1^+^CD11b^+^ myeloid-derived suppressor cells (MDSCs) represent the major cellular immunosuppressive network in tumors [4], [5]. Elimination of these immune suppressive cells has become a promising strategy to improve tumor immunotherapy.

TGFβ is a potent immunosuppressive cytokine which has the capacity to convert naïve CD4 cells into FoxP3-expressing Tregs [6]. TGFβ was reported to be responsible for the accumulation of Tregs in tumor by either expanding naturally occurring Tregs [7] or by converting naïve CD4 cells into induced Tregs [8]. In addition, it was reported that cell-cell contact inhibition of dendritic cells and T cells by Tregs was also mediated by TGFβ [9]. Furthermore, induction of MDSCs by tumor cells was at least partially mediated by TGFβ [10], [11]. Thus, TGFβ is generally believed to play a crucial role in the generation, accumulation and immunosuppressive effects of both Tregs and MDSCs in cancer.

The DNA alkylating agent cyclophosphamide (CY) is a commonly used cytotoxic medicine in the treatment of cancer [12]. In addition to its direct cytotoxic effect on cancer cells, CY also has a marked effect on immune cells, depending on the dose and timing of administration [13]. Recent work highlighted the immunostimulatory effects of low or metronomic dosing of CY in the boosting anti-tumor immune responses, based on promoting the maturation of dendritic cells, increasing the production of type I IFN, and induction of cytotoxic T cells and Th1/Th17 responses [13]. Intriguingly, CY was reported to preferentially eliminate Tregs, especially highly suppressive TNFR2^+^ Tregs present in the tumor environment [14], [15].

The highly tumorigenic and invasive mouse 4T1 mammary carcinoma model shares many of the characteristics of human breast cancer, particularly its ability to spontaneously metastasize to the lungs [16]. In this study, we initially examined the in vivo effects of 1D11, a neutralizing anti-TGFβ Ab, on the primary tumor growth and tumor infiltrating Tregs in the 4T1 model. We unexpectedly found that this anti-TGFβ Ab increased Tregs in the tumor-infiltrating CD4 cells, although the treatment inhibited tumor growth. To enhance the anti-tumor effect of 1D11, CY was combined with 1D11. Our study showed that this combination therapy turns out to be an effective chemoimmunotherapy regimen which may prove to be useful in the treatment of cancer patients.

## Materials and Methods

### Mice, cells and reagents

Female wild type 8 to 12 wk old Balb/c mice were provided by the Animal Production Area of the NCI (Frederick, MD). Foxp3/gfp KI mice were kindly provided by Dr. Yasmine Belkaid at NIAID, and maintained in the NCI-Frederick. BALB/c IFNγ^−/−^ mice were obtained from Jackson Laboratories. NCI-Frederick is accredited by AAALAC International and follows the Public Health Service Policy for the Care and Use of Laboratory Animals. Animal care was provided in accordance with the procedures outlined in the "Guide for Care and Use of Laboratory Animals" (National Research Council; 1996; National Academy Press; Washington, D.C.). Animal studies were approved by the Institutional Animal Care and Use Committee (IACUC) of National Cancer Institute (Frederick, MD).

4T1 breast cancer cells were obtained from ATCC (11/112003, lot No. 3306022 CRL-2539) and from Dr. Fred Miller (3/262003, Barbara Ann Karmacos Institute, Wayne State University School of Medicine) who firstly described this cell line [1]. 4T1 cells from Dr. Fred Miller was used in the spontaneous metastasis experimental format with surgical resection of the primary tumor, all other experiments were performed with 4T1 cells from ATCC. 4T1 cells from ATCC were examined with Molecular Testing of Biological Materials (MTBM) test (Animal Health Diagnostic Laboratory, NCI-Frederick) on 11/25/2003 and 10/27/2010, and 4T1 cells from Dr. Fred Miller was tested on 4/3/2003. CT26 colon cancer cell line, initially purchased from ATCC, was from the Laboratory of Experimental Immunology (NCI-Frederick) and was MTBM tested on 6/12/2007. The morphology, in vitro and in vivo growth rate and metastatic ability of cell lines were routinely monitored for the authentication. The latest Luminescence Mycoplasma Test (Cambrex MycoAlert, Animal Molecular Diagnostics Laboratory, NCI-Frederick) on both 4T1 cells and CT26 cells was performed on 10/11/11. Cell lines were cultured in RPMI-1640 medium supplemented with 10% FCS and 2 mmol/L glutamine at 37°C in a humidified incubator with 5% CO_2_.

Antibodies purchased from BD Biosciences (San Diego, CA) consisted of anti-CD3 (145-2C11), CD4 (GK1.5), CD16/CD32 (2.4G2), INFγ (XMG1.2). Foxp3 Staining Set (FJK-16s), anti-mouse TCRβ Ab (H57-597) and functional grade purified anti-mouse CD3e Ab (eBio500A2) were purchased from eBioscience (San Diego, CA). The anti mouse TGFβ monoclonal antibody, 1D11, which neutralizes all three isoforms of TGFβ, and an isotype-matched mouse IgG1 monoclonal antibody, 13C4, were provided by Genzyme Corp.

### Tumor cell inoculation and separation of tumor infiltrating lymphocytes (TILs)

4T1 tumor cells were injected into right mammary fat pads (thoracic No. 2 mammary glands) of recipient mice in single cells suspension with 50,000 cells in 0.2 ml PBS per mouse. After indicated times, tumors were excised, minced and digested in RPMI 1640 supplemented with 1 mg/ml collagenase IV and 0.1 mg/ml DNase I. The fragments were pushed through a 70-µm pore size cell strainer to create a single-cell suspension. In some experiments, two weeks after last treatment (60 days after initial tumor inoculation), tumor free mice after CY+1D11 treatment were re-inoculated with 4T1 cells (50,000) into the right mammary fat pads (thoracic No. 2 mammary glands), and the same number of CT26 colon carcinoma cells were s.c. injected to the left flank. Tumor size was calculated by the formula: (*Length* × *Width* ^2^)/2. “Survival” in the primary tumorigenesis studies represents the time to development of a 4 cm^3^ tumor or moribund, a humane endpoint that triggers euthanasia. To assess survival from metastatic burden in a spontaneous metastasis experimental format with surgical resection of the primary tumor, 40,000 4T1 cells in 40 µL of PBS were inoculated into the surgically-exposed left inguinal mammary fat pad of anesthetized mice. Primary tumors were surgically excised on day 12 as described previously [17]. Mice were monitored daily, and were euthanized when signs of morbidity from metastatic disease burden were evident. Lungs were fixed in 10% buffered formalin overnight and then washed with PBS, transferred to 70% ethanol and then embedded in paraffin, sectioned and stained with H&E.

### Treatment

Mice were treated by the following dose schedule: 1D11 or mouse IgG1 (13C4) were administered three times per week i.p at 0.1 mg in 0.2 mL PBS, starting 1 or 3 days after inoculation of 4T1 cancer cells. After 4 weeks, the three times weekly treatment was reduced to one. A single dose of CY was injected i.p. at 4 mg in 0.2 mL PBS by 3 days after cancer cell inoculation. For a reduced dose schedule, 1D11 or Mu IgG1 was i.p. administered at 0.1 mg, starting 3 days after 4T1 cancer cell inoculation. After 3 weeks, the three times weekly treatment was reduced to one. A single dose of CY was i.p. injected at 2 mg 3 days after cancer cell inoculation. For the spontaneous metastasis study with surgical resection of primary tumor, 1D11 or mouse IgG1 were administered three times per week i.p at 5 mg/kg for 2 wks starting 7 days after inoculation of 4T1 cells, followed by once a week treatment for the duration of the experiment. A single dose of 50 mg/kg CY was injected i.p. at day 14 after cancer cell inoculation.

### Flow Cytometry

After blocking FcR, cells were incubated with appropriately diluted antibodies. Acquisition was performed using a SLRII (BD Biosciences, Mountain View, CA) and data analysis was conducted using FlowJo software (Tree Star Inc., Ashland, OR). For intracellular cytokines staining, cells were re-stimulated with BD Leukocyte Activation Cocktail for 4 h. FACS analysis was gated on the live cells only by using LIVE/DEAD Fixable Dead Cell Stain Kit (Invitrogen Life Technologies). FACS analysis of TILs was gated on live CD45^+^TCRβ^+^(or CD3^+^) cells.

### Purification and *in vitro* culture of Treg cells

CD4^+^Foxp3/gfp^+^ Tregs were sorted from LNs and spleens of Foxp3/gfp KI mice using Cytomation MoFlo cytometer (Fort Collins, CO), yielding a purity of ∼98% Tregs. T-depleted spleen cells were used as APCs and irradiated with 3,000 R. Tregs were seeded into round-bottom 96-well plate at 2×10^4^ cells/well. The cells were stimulated with 2×10^5^ APCs/well plus 0.5 µg/ml of soluble anti CD3 Ab, with or without murine TNF (10 ng/ml, PeproTech, Rocky Hill, NJ), in the presence of medium alone or increasing concentration of recombinant human TGFβ1 (0.1∼1 ng/ml, R&D Systems, Minneapolis, MN) or 1D11 (1∼20 µg/ml). Cells were pulsed with 1 µCi [^3^H]thymidine (Perkin Elmer Life Sciences, Boston, MA) per well for the last 6 h of the 72-hour culture period.

### Statistical analysis

All data was compared and analyzed by two-tailed Student’s *t* test, except for the percent tumor free data and survival data which were compared and analyzed by Logrank test, using Graphpad Prism 4.0.

## Results

### Neutralization of TGFβ inhibits primary 4T1 tumor growth, but does not reduce the proportion of Foxp3^+^ Tregs in tumor infiltrating CD4 subset

**Figure 1:**
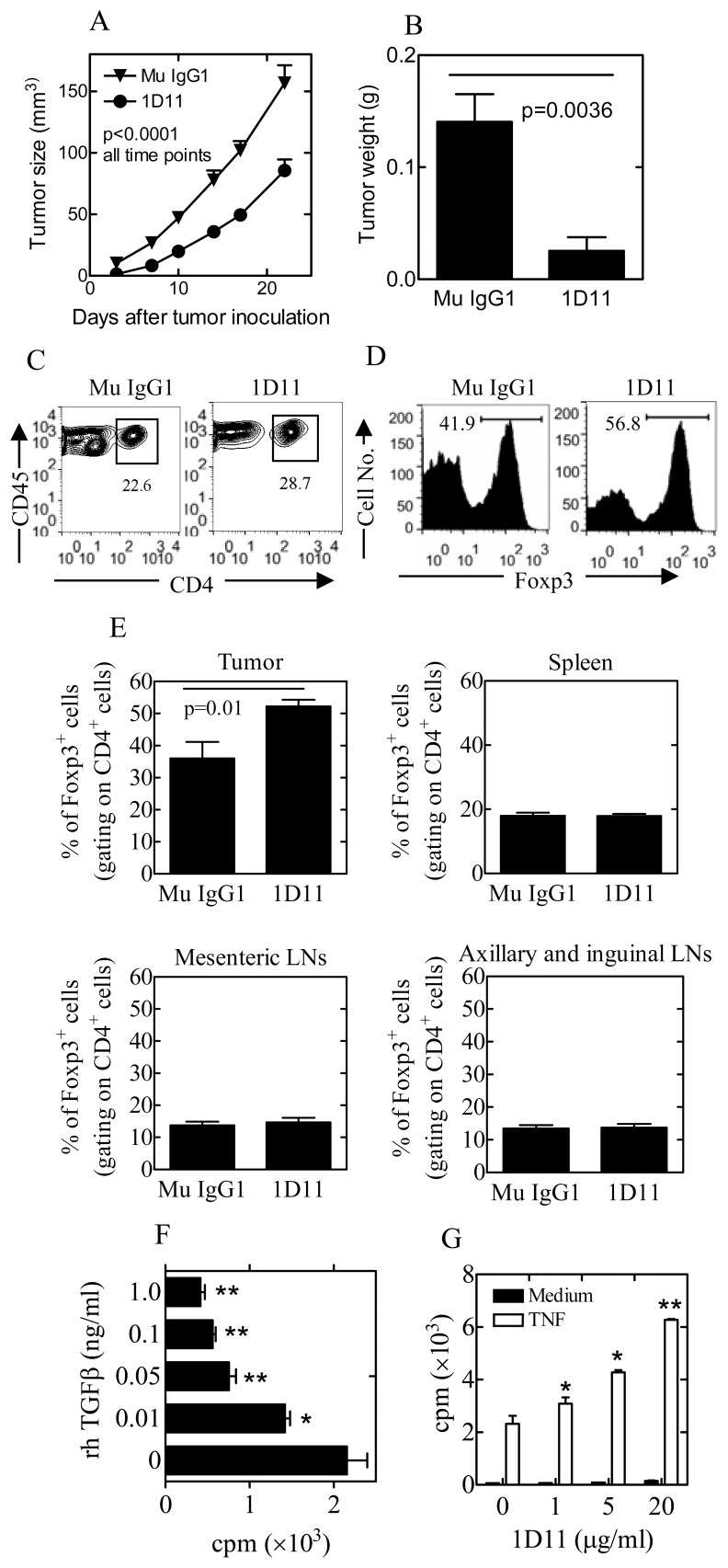
Effects of 1D11 on 4T1 tumor growth and on the expansion of Tregs in vivo and in vitro. (A-D) Mice were treated with 0.1 mg 1D11 or mouse IgG1 (i.p., 3×week), starting at day 1 of tumor inoculation. (A) Kinetics of tumor growth. (B) Weight of tumors isolated at 14 days after inoculation. (C-E) Effect of 1D11 on Tregs. CD4 cells and Tregs was analyzed with FACS at 14 days after tumor inoculation. (C, D) Typical FACS analysis of CD4 cells and Tregs. Number represents the percentage of CD4^+^ cells in total tumor infiltrating CD45^+^ leukocytes (C) or Foxp3^+^ cells in intratumoral CD45^+^CD4^+^ cells (D). (E) Summary of proportion of Foxp3^+^ cells in CD4 cells present in the tumor, spleen, mesenteric LNs and axillary/inguinal LNs (N = 3∼7). (F-G) TGFβ inhibits, while 1D11 promotes, proliferation of Tregs in vitro. (F) CD4^+^Foxp3/gfp^+^ Tregs were stimulated in the presence of TNF with increasing concentrations of rhTGFβ1. (G) Tregs were stimulated in the presence of TNF or medium alone with increasing concentration of 1D11. After incubation for 72 hours, the proliferation of Tregs was determined by [^3^H] thymidine incorporation. By compared with respective control (without rhTGFβ1or 1D11), *p<0.05, ** p<0.01. N = 3. The data are representatives of three separate experiments with same results.

To examine if anti-tumor effect of the anti-TGFβ Ab 1D11 [17] was based on the elimination of Treg accumulation in the tumor, mice inoculated with 4T1 tumor cells were treated with 1D11 or Mu IgG1. The treatment was started at 1 day after tumor inoculation for early blockage of TGFβ. Results showed that 1D11 treatment markedly inhibited the growth of primary 4T1 tumor (p<0.0001, Fig 1A), resulting in a smaller tumor mass (p<0.01, Fig 1B). The proportion of CD4^+^ cells in the tumor infiltrating CD45^+^ leukocytes was 28.7% in 1D11-treated mice, markedly higher than that in control IgG1- treated mice (22.6%, P<0.05, Fig C). Surprisingly, the proportion of Tregs present in tumor infiltrating CD4 cells in 1D11-treated mice was markedly increased, as compared with mice treated with control IgG1 (p = 0.01, Fig 1D-E). In contrast, the proportion of Foxp3^+^ Tregs in the spleen, mesenteric LNs and axillary/inguinal LNs was not increased (Fig 1E).

This result suggest that neutralization of TGFβ might promote proliferation of Tregs in the tumor inflammatory environment. To test this, we examined the effect of TGFβ and anti-TGFβ Ab on the proliferation of Tregs in vitro. Previously we showed that the profound hyporesponsiveness of Tregs to TCR stimulation in vitro could be overcome by exogenous TNF [18], a major proinflammatory mediator elevated in the tumor microenvironment with the capacity to promote growth and metastatic spread of cancer [19]–[21]. We now observed that the proliferation of Tregs in the presence of TNF was potently inhibited by TGFβ, in a dose-dependent manner (0.01∼1 ng/ml, p<0.01∼0.05, Fig 1F). 1D11 (1∼20 µg/ml) by itself did not promote the proliferative response of Tregs to TCR stimulation, however, this antibody was able to markedly promote the proliferation of Tregs in the presence of TNF (p<0.01∼0.05, Fig 1G). Therefore, the increased proportion of Tregs in 4T1 tumor after 1D11 treatment is likely caused by the abrogation of the inhibitory effect of TGFβ on Tregs in tumor environment.

### Combination of 1D11 and CY inhibited the development of 4T1 tumor

**Figure 2:**
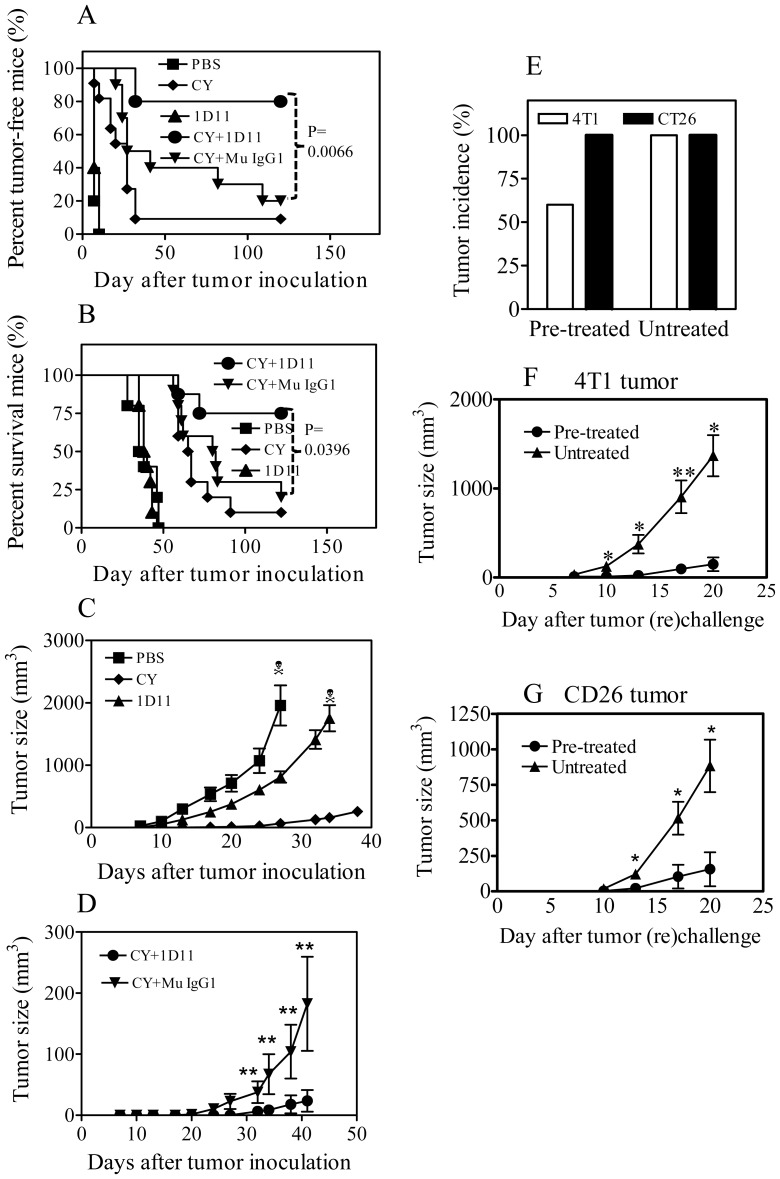
1D11 in combination with CY potently inhibits the development of mouse 4T1 tumor and induces anti-tumor immunity. Three days after tumor inoculation, the mice were i.p. treated with single dose of CY (4 mg) or 1D11 (0.1 mg, 3×week), or combination of CY and 1D11 or mouse IgG1. (A) Percent tumor-free mice (%). (B) Survival of tumor inoculated mice. (C) Tumor size in groups treated with PBS, or CY alone or 1D11 alone. (D) Tumor size in groups treated with CY+1D11 or CY+Mu IgG1. Two weeks after last 1D11 treatment (60 days after initial tumor inoculation), the tumor-free mice (designated as pre-treated) were re-inoculated with 4T1 cells into the right thoracic mammary fat pad, and CT26 cancer cells were inoculated (s.c.) into the left flank. For comparison, age- and gender-matched normal Balb/c mice (designated as untreated) were inoculated with 4T1 and CT26 tumor cells in the same manner. (E) Incidence of 4T1 and CT26 tumor development on day 18 after tumor inoculation. (F) Growth of 4T1 tumor and (G) growth of CT26 tumor. Data shown in C, D, F and G are means±SEM (N = 5∼10). Comparison of two groups: * p<0.05; **p<0.01. The data are representatives of three separate experiments with similar results.

**Figure 3:**
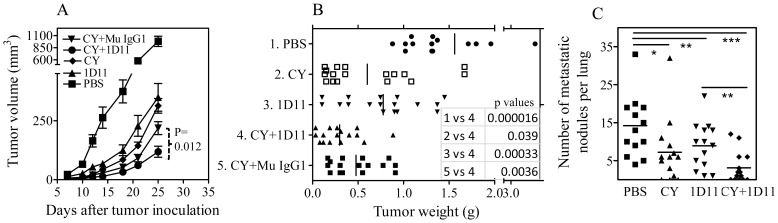
Effect of combination of 1D11and reduced dose of CY on primary tumor growth and pulmonary metastasis. Three days after tumor inoculation, the mice were i.p. treated with single dose of CY (2 mg) or 1D11 (0.1 mg, 3×week), or combination of CY and 1D11 or mouse IgG1. Mice were sacrificed ∼4 wks after tumor inoculation. (A) Kinetics of tumor growth. The data are representatives of three separate experiments with similar results (N = 10, data shown as means±SEM). (B) Weight of tumors after 4 wks of inoculation (N = 17, pooled from two separate experiments). (C) The number of grossly visible metastatic nodules in the lung (N = 14, pooled from two separate experiments). Comparison of indicated groups, * p<0.05, ** p<0.01, *** p<0.001.

Although 1D11 suppressed the growth of 4T1 tumors, it failed to completely control their growth, which may be attributable to the expansion of Tregs in the tumor. A therapeutic with the capacity to eliminate tumor infiltrating Tregs may enhance the anti-tumor action of 1D11. It was reported that tumor infiltrating TNFR2^+^ highly suppressive Tregs could be eliminated by CY [15]. We therefore examined the effect of combination treatment of 1D11 and CY. A single dose of CY (4 mg) was administered to mice 3 days after inoculation of tumor cells, in order to reflect a more therapeutic setting. The 1D11 treatment was started same day of CY administration. All mice (100%) treated with 1D11 alone developed tumor (Fig 2A) and died at week 7, without any survival benefit as compared with the untreated group (p>0.05, Fig 2B), although this treatment consistently inhibited tumor growth (Fig 2C). CY treatment alone markedly delayed the development of solid tumor (p<0.0001, Fig 2A) and increased the survival of tumor-challenged mice (p<0.0001, Fig 2B). Very interestingly, 80% of mice in the group treated with 1D11+CY did not develop 4T1 tumor at all; in contrast, only 10% and 20% of mice were tumor free after CY treatment alone, or CY+Mu IgG1 treatment (p<0.01, Fig 2A). All mice that failed to develop tumors remained alive more than 100 days after tumor inoculation (Fig 3B). The tumor volume in the CY+1D11 combination treatment group was also smaller than in the CY alone treatment group (p<0.01, Fig 2C-D), while CY+Mu IgG1 treatment showed no difference from CY treatment alone (P>0.05, Fig 2C-D).

### Tumor-free mice after CY+1D11 treatment are more resistant to tumor re-challenge

To examine whether the tumor-free mice developed 4T1 tumor-specific immunity, those mice surviving after CY+1D11 treatment were re-inoculated with 4T1 tumor cells and the same number of mouse CT26 colon cancer cells on the contralateral flank. As a control, normal Balb/c mice were also inoculated with 4T1 cells and CT26 cells in the same manner. All mice (100%) in the control group developed measurable 4T1 and CT26 tumor by day 13 after inoculation (Fig 2E). Although all mice in CY+1D11-pretreated group developed CT26 tumor by day 13, only 60% developed 4T1 tumor by day 18 (Fig 2E). Furthermore, the size of 4T1 and CT26 tumors in CY+1D11 pre-treated mice was markedly smaller as compared with that in normal control mice (p<0.01∼0.05, Fig 2F-G). These data indicate that tumor-free mice after 1D11 and CY treatment at least partially developed specific resistance to the 4T1 tumor that they had previously rejected. Further, CY+1D11 pre-treated mice also developed some non-specific resistance to challenge with a different tumor.

### Dose-reduced 1D11+CY treatment also inhibits 4T1 tumor growth and lung metastasis

Although our original treatment regimen achieved an optimal anti-tumor effect, it did not allow us to examine Tregs and other TILs, since the majority of mice did not develop tumor at all. Therefore, we administered lower doses of both 1D11 and CY in order to allow tumor growth for analysis. The results show that 90% of mice developed tumor after treatment with reduced doses of CY and 1D11. This treatment regimen also markedly inhibited the growth of primary tumor (p<0.05∼0.001), as shown in Fig 3A-B. We were able to confirm previous observations [11], [17], [22] that 1D11 by itself inhibited lung metastasis (Fig 3C, p<0.01). Moreover, such anti-metastatic activity of 1D11 was markedly enhanced by combination with CY (Fig 3C, p<0.001∼0.01, as compared with PBS or 1D11 alone).

### Combination therapy of 1D11+CY promotes infiltration of IFNγ-producing T cells into the tumor

**Figure 4:**
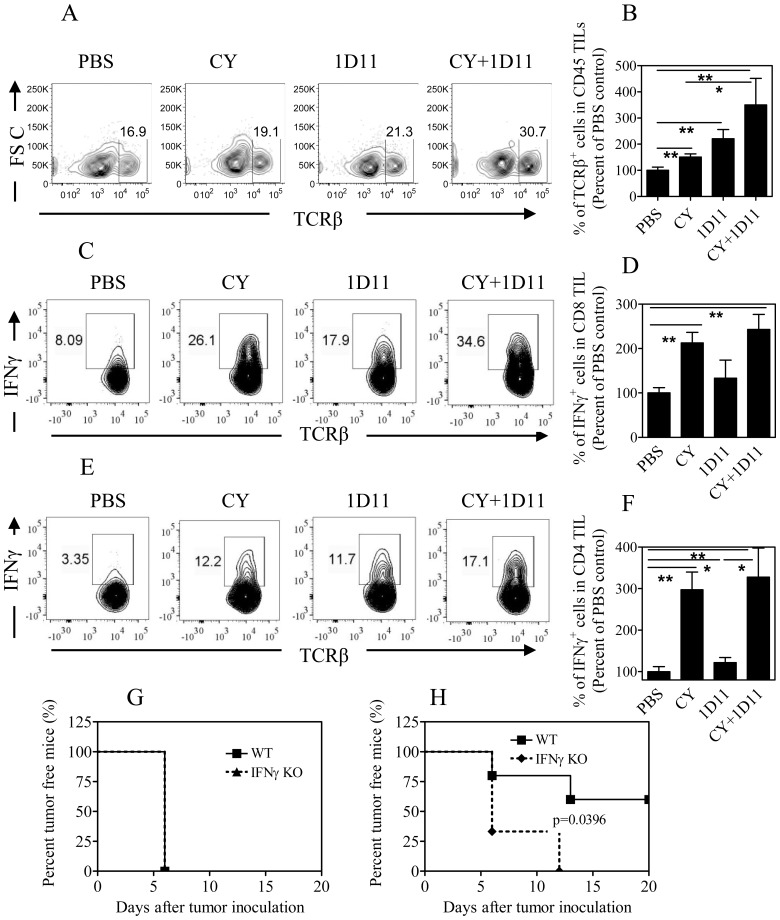
Combination treatment of 1D11 and CY promotes tumor infiltration of IFNγ-producing T cells. Four weeks after 4T1 tumor inoculation, cell suspension was prepared from tumor tissues. (A-B) Proportion of TCRβ^+^ T cells in total tumor infiltrating CD45^+^ leukocytes. Typical flow plots are shown in (A), and summary of data from three separate experiments is shown in (B, Means±SEM, N = 14∼20). (C-F) IFNγ expression by CD8 and CD4 TILs. Intracellular expression of IFNγ was analyzed by FACS, gating on live CD45^+^TCRβ^+^CD8^+^ cells (C-D) or CD45^+^TCRβ^+^CD4^+^ cells (E-F). Data shown are typical FACS plots (C, E) and summary of data (D, F) from three separate experiments (Means±SEM, N = 9). Comparison of indicated groups, * p<0.05, ** p<0.01. **(**G, H**)** Normal WT Balb/c mice and IFNγ KO mice were inoculated with 4T1 cells and treated with 1D11+CY in the same manner. (G) Incidence of 4T1 tumor in mice treated with PBS. (H) Incidence of 4T1 tumor in mice treated with 1D11 and CY. Data are shown as percent tumor free mice (%, KO mice N = 3, WT mice N = 5), which are representatives of two separate experiments with similar results.

Since tumor-free mice after CY+1D11 treatment developed partial 4T1 tumor-specific resistance, we hypothesized that T cells should be mobilized and activated. Indeed, 1D11 treatment alone, and CY treatment alone to a lesser extent, increased T cell infiltrating the tumor (p<0.01, Fig 4A-B). Tumor-infiltrating T cells were further increased after combination treatment and were >3-fold and >2-fold greater than in tumors of mice treated with PBS or CY alone (p<0.05∼0.01, Fig 4A-B). Importantly, combination treatment with CY+1D11 markedly increased IFNγ production by both CD8 and CD4 T cells (p<0.01, Fig 4C-F). In contrast, Mu IgG1+CY treatment resulted in a lower proportion of IFNγ-producing T cells (data now shown) than that treated with 1D11+CY, suggestive that the effect of 1D11 is not based on a non-specific action of IgG1. This effect of combination treatment is mainly attributed to the ability of CY in stimulating this Th1 cytokine production, since CY treatment alone resulted in the ∼2-fold and ∼3-fold increase of IFNγ-producing cells in CD8 subset and CD4 subset (p<0.01), while 1D11 treatment only resulted in 33% and 22% increase of IFNγ-producing CD8 and CD4 cells, respectively. Therefore, 1D11 is mainly responsible for the infiltration of T cells, while CY is major driving force for the Th1 polarization. Thus, these two therapeutics together complement each other, resulting in the massive infiltration of IFNγ-producing T cells into the tumor.

To evaluate the role of IFNγ in the anti-tumor effect of the combination treatment of CY+1D11, we examined its effect in 4T1 tumor-bearing IFNγ KO mice. As can be seen in Fig 4G, there was no difference in the tumor development in both WT and IFNγ KO mice. However, although the combination treatment resulted in 60% of WT mice being tumor-free by day 20, tumors developed in all IFNγ KO mice treated with CY+1D11 by day 12 (p<0.05, Fig 4H). The tumor incidence in IFNγ KO mice treated with CY+1D11 had no significant difference as compared with that in WT or IFNγ KO mice without treatment (p>0.05). Therefore, the anti-tumor effect of the combination therapy is at least partially dependent on IFNγ.

### Combination therapy of 1D11 and CY reduces the number of MDSCs and promotes re-differentiation of MDSCs

**Figure 5:**
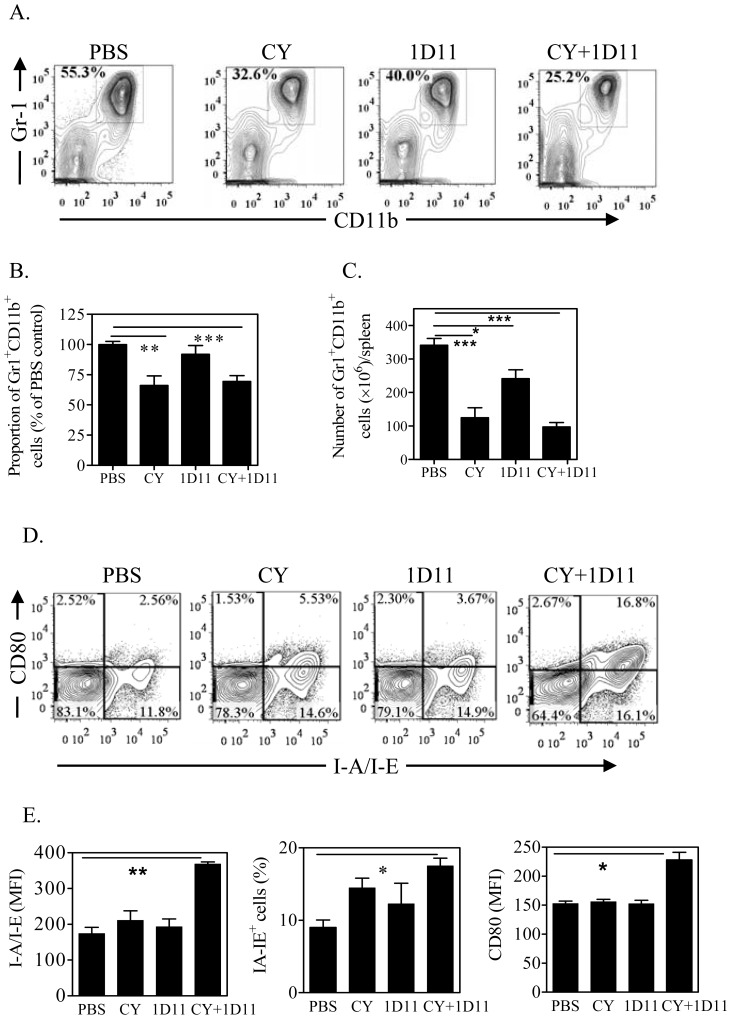
Combination treatment of 1D11 and CY reduces the number of splenic MDSCs and promotes their re-differentiation. Four weeks after 4T1 tumor inoculation, cell suspensions were prepared from spleen and MDSCs were analyzed by FACS, gating on live CD45^+^ cells. (A-B) Proportion and number of Gr1^+^CD11b^+^ cells in the spleens. Typical FACS plots are shown in (A, gating on total live splenic CD45^+^ cells) and summary of data pooled from three experiments are shown in (B, percent of PBS control group, Means±SEM, N = 10∼13). (C) Absolute number of Gr1^+^CD11b^+^ cells in the spleen (N = 11, pooled from two separate experiments). (D-E) Expression of I-A/I-E and CD80 on Gr1^+^CD11b^+^ splenic cells. (D) Typical FACS plots (gating on Gr1^+^CD11b^+^ cells) and (D) summary of data pooled from three separate experiments (Means±SEM, N = 9). Comparison of indicated groups, *p<0.05, ** p<0.01, ***p<0.001.

Since we had shown that 1D11 treatment increases intratumoral Tregs, and CY was reported to reduce Tregs, we predicted that CY would abrogate 1D11-driven expansion of Tregs. However, unexpectedly, we did not find any change in intratumoral Tregs following combination therapy (data now shown). We therefore looked for alternative mechanisms. The mouse 4T1 tumor model is characterized by the accumulation of MDSCs in the spleen which causes splenomegaly [11]. Combination treatment with 1D11 and CY markedly reduced the spleen weight and cellularity of 4T1 tumor-bearing mice (p<0.01, data not shown), suggesting that the combined treatment may reduce the number of splenic MDSCs. Indeed, the proportion of Gr1^+^CD11b^+^ MDSCs in the spleen from mice treated with CY, CY+1D11 was markedly reduced by 43% and 54% (p<0.01, Fig 5A-B). The reduction of MDSCs was mainly attributable to the CY treatment, and may be secondary to the reduced tumor burden. In the tumor, CY and 1D11+CY similarly reduced the proportion of Gr1^+^CD11b^+^ cells in CD45^+^ tumor infiltrating leukocytes (data not shown). The cell number of Gr1^+^CD11b^+^ cells in the spleen of 4T1 tumor-bearing mice was markedly reduced by the treatment of CY or 1D11 alone or their combination (p<0.05∼0.001, Fig 5C). Importantly, combination treatment, but not either therapeutic alone, markedly enhanced the expression of MHC II (% and MFI) and co-stimulatory CD80 (MFI) on splenic Gr1^+^CD11b^+^ cells (p<0.01∼0.05, Fig 5D-E). Thus, this combination regimen not only reduced the number of MDSCs, but also induced the maturation and/or differentiation of myeloid cells in tumor-bearing mice.

### Combination therapy of CY and 1D11 prolonged the survival of mice with spontaneous lung metastasis

**Figure 6:**
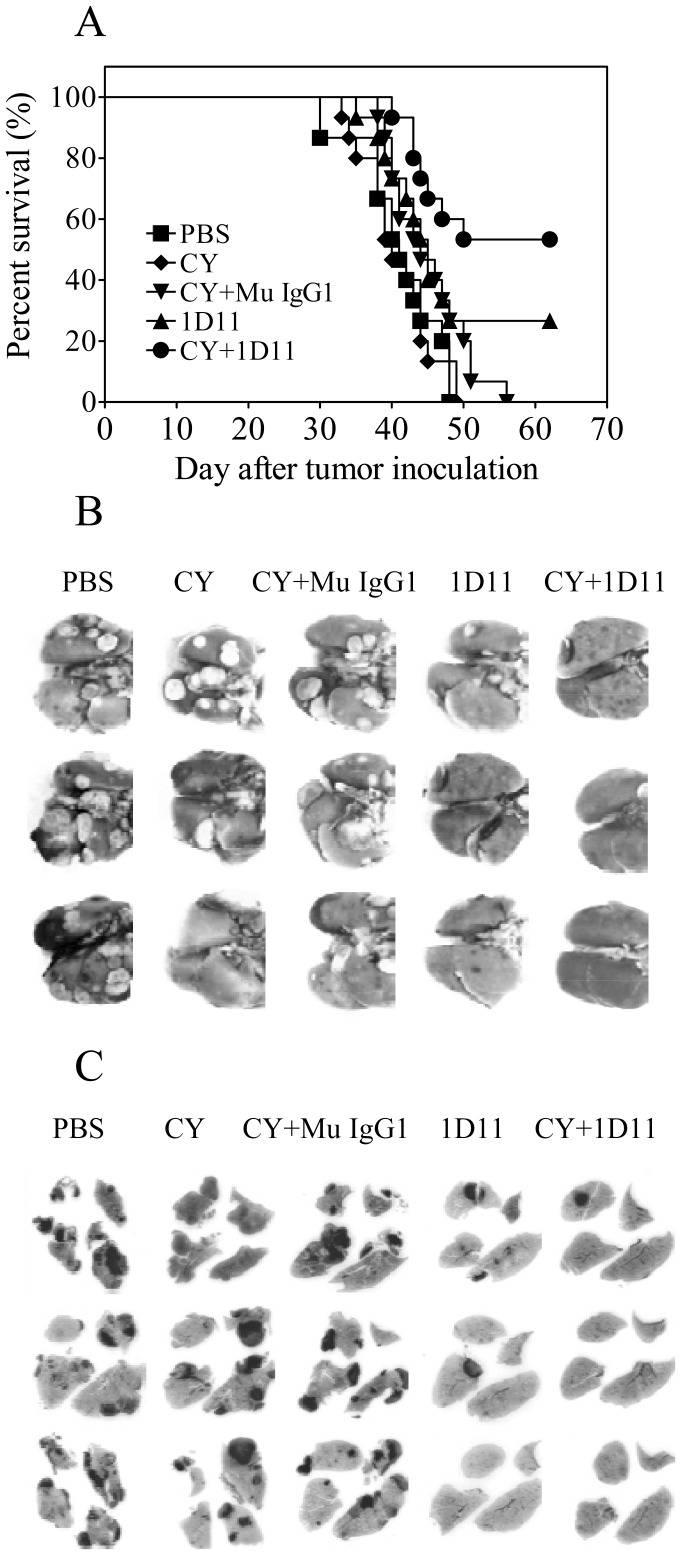
Combination treatment of 1D11 and CY increases survival of mice with 4T1 lung metastasis in an orthotopic implantation/resection format. 4T1 cells were inoculated into the mammary fat pad and primary tumors were surgically removed at day 12 after inoculation. 1D11 or Mu IgG1 (13C4) control antibody (5 mg/Kg, i.p.) were administered three times per week for the first two weeks and followed by once a week, starting from 7 days after tumor inoculation. A single subtherapeutic dose of CY (50 mg/Kg, i.p.) or vehicle was given on day 14, two days following surgical resection of the primary tumor. The therapeutics was treated alone or combined as indicated. (A) Survival curves for the different treatment groups. Survival curves are significantly different between the groups (p<0.0007; Log-Rank (Mantel-Cox) Test). (C) Representative images from lungs at gross and (D) in histological cross-section (H&E stained).

We further utilized the 4T1 model in a format where the primary 4T1 tumor was surgically excised and mouse survival is driven by metastatic lung disease [17]. As shown in Figure 6, mice in PBS treated group had a median survival time of 41 days. Neither treatment with Mu IgG1 (median survival 42 days, data not shown), nor the subtherapeutic dose of CY (median survival 40 days) had any effect on survival. 1D11 treatment alone showed a trend toward increased survival (median survival 45 days; p =  0.07 vs PBS control). However, the combination treatment with 1D11 and CY markedly increased survival (p<0.003, as compared with PBS treatment alone), with more than half the mice still alive at the end of the experiment on day 62. Primary tumor weights at the time of surgical resection were not significantly different between the groups (data not shown), indicating that the short (5 day) neoadjuvant treatment with 1D11 did not affect the primary tumor in this experimental format. Therefore, combination therapy with 1D11 and CY can directly inhibit metastasis and significantly increase overall survival in a clinically more realistic treatment setting where morbidity and death are driven by lung metastatic burden.

## Discussion

TGFβ is a pleiotropic cytokine that plays a key role in the interplay of tumor cells and other cells in the tumor environment [23]. High levels of TGFβ are expressed in many advanced human cancers and circulating levels of TGFβ frequently correlate with increased metastasis and poor prognosis [24], [25]. As a potent immunosuppressive cytokine, TGFβ inhibits the activation of cytolytic T lymphocytes (CTL), natural killer cells (NK) and macrophages, and promotes differentiation of Tregs [6] and MDSCs [10], and consequently suppresses immune surveillance against the tumor. However, TGFβ as an inhibitory cytokine also has tumor suppressor function. It inhibits cell cycle progression and consequently increases apoptosis of cancer cells, suppresses the expression of growth factors, cytokines and chemokines which are critical for tumor development [23], [26]–[28].

Thus, TGFβ has dual and biphasic effects in tumor development, and this complex nature of TGFβ in cancer biology poses the challenge for the application of TGFβ inhibitor as a sole therapeutic. Combination with other therapeutics has the potential to reinforce the beneficial anti-tumor effects, while minimizing the undesirable effects of a TGFβ inhibitor. Our study clearly shows that CY is one such chemotherapeutic. This combination treatment likely targets multiple cellular and molecular events simultaneously. Nevertheless, the activation of anti-tumor immune responses contributes substantially to the anti-tumor effect , for the reason that: 1) the combination treatment resulted in a massive infiltration of IFNγ-producing cells to the tumor; 2) anti-tumor effect of the combination therapy was reduced in IFNγ KO mice; 3) the number of splenic MDSCs was markedly reduced and the residual MDSCs showed a more mature phenotype; 4) tumor-free mice after CY+1D11 treatment developed long term anti-tumor immunity.

We anticipated that CY treatment would reduce the number of Tregs, as previously reported [29]. However, four weeks after treatment with CY, we did not observe a significant reduction of Tregs in tumor infiltrating CD4 cells. Recent studies revealed that CY only transiently reduced the number of Tregs in tumor-bearing mice. Reduction of Tregs occurred on 3∼7 days post treatment, and after that, the number of Tregs restored and rebounded to even higher levels [30]–[32]. In human cancer patients, treatment with metronomic dose of CY also only resulted in a transient reduction of Tregs [33]. Although we did not observe the reduction of Tregs after 4 weeks of CY treatment, presumably due to the recovery of Tregs after transient depletion, the number of MDSCs was markedly reduced in both spleen and tumor. It was reported that CY treatment led to a transient surge of "MDSCs" in tumor-free normal mice [34], [35], however, these CY-induced "MDSCs" were phenotypically different from MDSCs found in tumor bearing mice [36]. Recently, it was reported that CY, in combination with IL-12, depleted immunosuppressive MDSCs and at same time induced inflammatory myeloid cells, resulting in inhibition of tumor growth in a mouse model of cancer [35]. In our study, CY treatment alone potently reduced the proportion and number of Gr1^+^CD11b^+^ cells in the spleen (p<0.01, Fig 5) and tumor (data not shown). Importantly, the combination treatment with CY and 1D11, but not therapeutics alone, resulted in a markedly higher expression of MHC II and CD80 on CD11b^+^Gr1^+^ cells (Fig 5). Further study is needed to clarify if these myeloid cells are same as previously described inflammatory myeloid cells. Presumably, in mice treated with 1D11+CY, the 4T1 tumor-specific immune responses were mediated by IFNγ-producing T cells, and non-specific anti-cancer immune responses were mediated by inflammatory myeloid cells. TGFβ is also able to induce Foxp3 expression on CD8^+^ Tregs [37], and this subset of suppressor cells contributed to the immunosuppression in certain types of tumor such as prostate cancer [38]. Since CY was reported to inhibit the generation and function of CD8^+^ Tregs, it is possible that the combination of 1D11 and CY further eliminate CD8^+^ Tregs in tumor bearing mice. However, we did not find any CD8^+^Foxp3^+^ Tregs in the tumor tissues or peripheral lymphoid tissues of 4T1 breast cancer bearing mouse.

Although TGFβ is able to convert naïve CD4 cells into FoxP3-expressing induced Tregs (iTregs) [6], this cytokine actually restrains the proliferative expansion of pre-existing naturally occurring Tregs (nTregs) (Fig 1). Therefore, our data strongly supports a dual role of TGFβ in Treg activity, e.g., promoting differentiation of induced Tregs while inhibiting the proliferative expansion of naturally occurring Tregs. This may explain the paradoxical observation in mice with conditional deletion of TGFβ receptor I (TβRI) in T cells: Although the appearance of Foxp3^+^ Tregs in neonatal mouse thymus was delayed, beginning 1 week after birth, there was an accelerated expansion of thymic Tregs in this mouse [39]. Further, the increase of Tregs in tumor infiltrating CD4 subset after 1D11 treatment (Fig 1) negate the possibility that accumulation of Tregs in tumor was caused by the expansion of Tregs induced by TGFβ [7], or conversion of naïve CD4 cells into iTregs by TGFβ [8]. We favor the idea that other mediators such as TNF may attribute to the proliferative expansion of Tregs in tumors [14]. This notion is supported by a recent study showing that TNF-TNFR2 interaction is responsible for the accumulation of Tregs in B16F10 melanoma mouse model [19].

A previous report showed that the combination of CY with an anti-TGFβ receptor II antibody had an additive effect in the suppression of primary tumor growth and lung metastasis in mice bearing EMT6 mammary cancer [40]. However, our study differs in a number of significant ways. We showed that the development of primary tumor could be completely inhibited, with a clear benefit of long term tumor-free survival, by the treatment of CY + anti-TGFβ (1,2,3) antibody (1D11) (Fig 2), while treatment with CY + anti-TGFβ receptor II antibody only resulted in a partial inhibition [40]. Importantly we found that mice treated with the combination therapy were resistant to tumor-rechallenge, suggesting the development of durable anti-tumor immunity. Mechanistically, we found a novel synergistic effect of TGFβ antagonism and CY on the stimulation of intratumoral infiltration of IFNγ-producing T cells. The possibility that antibody-driven neutralization of ligand may be more efficacious at blocking the TGFβ pathway than antibody mediated receptor blockade needs to be further explored.

In our studies, Mu IgG1 13C4 by itself or in combination with CY had a consistent inhibitory effect on 4T1 tumor growth (Fig 2 and Fig 3). This therapeutic effect of 13C4 is presumably attributable to the Fc-mediated effector functions of IgG1 [41]. Nevertheless, anti-tumor effect of 1D11 and 1D11+CY was markedly greater than that of 13C4 and 13C4+CY, indicative of the net effect of neutralization of TGFβ.

Taken together, our study showed a commonly used chemotherapeutic CY was able to enhance the anti-tumor effect of TGFβ inhibitor, resulting in the potent inhibition in the development of 4T1 mouse mammary carcinoma. Although multiple mechanisms may underlie the anti-tumor effect of combination therapy of CY and TGFβ inhibitor, this efficacy is in part due to improvement in the quality of anti-tumor immunity in both adaptive and innate arms. This combination regimen thus represents a successful approach to the chemoimmunotherapy of primary and metastatic cancer.
